# The Rapid Evaluation of Down Syndrome With Quantitative Fluorescence Polymerase Chain Reaction (QF-PCR): A Pilot Study Among the Population in Eastern Uttar Pradesh, India

**DOI:** 10.7759/cureus.59241

**Published:** 2024-04-28

**Authors:** Maneesha Upadhyay, Nitish K Singh, Ashish Ashish, Meenakshi Upadhyay, Ankur Singh, Royana Singh

**Affiliations:** 1 Anatomy, Institute of Medical Sciences, Banaras Hindu University, Varanasi, IND; 2 Surgery, Institute of Medical Sciences, Banaras Hindu University, Varanasi, IND; 3 Pediatric Medicine, Institute of Medical Sciences, Banaras Hindu University, Varanasi, IND

**Keywords:** marker, str, karyotype, qf-pcr, down syndrome

## Abstract

Background and objective

Down syndrome (DS) is characterized by the presence of an additional chromosome; it is a typical chromosomal disorder causing intellectual disability in individuals. The diagnostic process for DS often involves conventional karyotyping, which can be time-consuming. Trisomy 21 and other chromosomal abnormalities may now be quickly and accurately diagnosed using quantitative fluorescence polymerase chain reaction (QF-PCR). In light of this, this study aimed to investigate chromosomal abnormalities in DS using conventional karyotyping and QF-PCR among the population in eastern Uttar Pradesh, India.

Methods

Blood samples from 40 individuals with clinically diagnosed DS were collected. Conventional karyotyping involved standard cytogenetic techniques, while QF-PCR utilized DNA extraction and analysis with chromosome-specific short tandem repeat (STR) markers.

Results

Various distinct physical characteristics were observed in the DS individuals, such as mongoloid slant and low-set ears. Karyotyping and QF-PCR analyses revealed different chromosomal configurations associated with DS trisomy 21, with additional chromosomal abnormalities found in some individuals, including partial monosomy 18 and mosaic trisomy 21. However, in a few cases, neither karyotyping nor QF-PCR revealed any abnormalities.

Conclusions

The study demonstrated that QF-PCR is a reliable and rapid method for diagnosing DS, providing results within 24 hours. This approach allows for the simultaneous diagnosis of a large number of samples and reduces the time required to obtain results. In the diagnostic procedure for DS, we believe QF-PCR will prove to be a useful tool. Furthermore, therapeutic interventions based on their clinical traits and molecular karyotyping can enhance the quality of life of people with DS.

## Introduction

Down syndrome (DS) is the most prevalent chromosomal issue and neurodevelopmental disorder linked to intellectual disability, affecting one in every 800 births worldwide [1]. Its prevalence varies among nations due to various social, cultural, and economic factors, including the average maternal age of conception, prenatal testing, and access to abortion [2]. The phenotypic characteristics are significant in the context of the present study. Phenotype characterization plays an important role in aiding pediatricians and other healthcare professionals in the diagnosis of DS. Based on the genetic identification of DS, it appears that chromosome 21 carries an additional copy in every cell (trisomy 47, XX, +21 or 47, XY, +21), which is brought on by meiotic nondisjunction during gametogenesis [3,4,5].

Karyotyping and quantitative fluorescence polymerase chain reaction (QF-PCR) can both be performed to diagnose prenatal chromosomal disorders [6]. Conventional karyotype analysis is considered the gold standard for diagnosing chromosomal abnormalities, but it can be time-consuming [7]. On the other hand, QF-PCR has been demonstrated to be a speedy, effective, and trustworthy method [8]. QF-PCR usually requires only two to three days to examine chromosomes 13, 18, and 21, as well as the sex chromosomes [9]. Several studies from various countries have described the diagnostic utility of QF-PCR in trisomy 21 [10,11,12]. However, very few studies have explored the application of QF-PCR in DS among Indian patients [13,14,15]. This pilot cross-sectional study aims to gather useful data on the applicability and validity of QF-PCR as a quick diagnostic method for DS in children, including neonates, among our local community and give insights into prospective advances in the assessment and therapy of this population.

## Materials and methods

Participant selection

The study involved the selection of 40 blood samples from individuals clinically diagnosed with DS by a pediatrician from the Department of Pediatric Medicine. Based on their clinical features, the children were randomly assigned to either conventional karyotyping or QF-PCR groups

Study design and setting

This was a cross-sectional study conducted at the Department of Pediatric Medicine and Anatomy, Institute of Medical Sciences, Banaras Hindu University, Varanasi, Uttar Pradesh, India.

Inclusion and exclusion criteria

All children with clinical features of DS were included for QF-PCR and conventional karyotyping. Those children who did not show any clinical features of DS were excluded.

Ethical clearance

The study received ethical clearance from the Institutional Ethical Committee of the Institute of Medical Sciences, Banaras Hindu University (ref. no. Dean/2022/EC/3360).

Study sample size

The study was conducted using a systematic random sampling approach among individuals with DS who attended the pediatric outpatient department (OPD) between January 2022 and December 2022. We assumed the following variables in terms of various parameters: standard normal variant for 5% level of significance for two-tailed tests = z α/2 = 1.96, the proportion of DS per live births in India = 12/600 = 0.02 (based on the Department of Paediatric OPD data of Sir Sunderlal Hospital, Banaras Hindu University Varanasi), and non-response rate = r = 0.05. Ultimately, a total sample size of 162 individuals was calculated. However, to undertake the pilot study, 25% of the total sample was selected, resulting in a sample size of 41 individuals.

Sample collection procedure

Karyotyping

Peripheral venous blood samples were collected from participants, and culture tubes were filled with HyClone RPMI 1640 liquid (Gibco, Life Technologies, Grand Island, NY). Which was supplemented with L-glutamine, 10% fetal bovine serum, penicillin-streptomycin solution, and phytohaemagglutinin. Each sample was cultured for 72 hours in a CO_2_ incubator with parallel cultures and labels. Colchicine was added to each culture tube and incubated for one hour. Cell suspensions were collected after centrifugation, and 0.075M KCl hypotonic solution was added. Conroy's fixative was applied to the pellet. After staining slides with 1% Giemsa, each slide was observed under Applied Spectral Imaging (Advanced Chromosome Analysis, Olympus). At least 20 metaphases were examined for chromosomal abnormalities or mosaicism. Karyotypic results were generated by using an International System for Human Cytogenetic Nomenclature (2016) [16,17].

Quantitative Fluorescence Polymerase Chain Reaction (QF-PCR)

DNA was extracted from blood samples using the Qiagen QIA amp DNA Blood Mini Kit (cat No. 51304). DNA was assessed using the NanoDrop technique following the 260/280 ratio. PCR used highly polymorphic short tandem repeat (STR) markers and labeled primers to amplify DNA fragments. Compact v3 Mix of PCR Activator (20µL) was mixed with 5µL of genomic DNA in each PCR reaction tube [18] and followed the PCR temperature conditions as detailed in Table 1.

**Table 1:** PCR cycle and temperature condition PCR: polymerase chain reaction

Temperature	Time	Cycle
95 °C	15 minutes	1
94 °C	30 seconds	27
58 °C	90 seconds	
72 °C	90 seconds	
72 °C	30 seconds	1

**Figure 1:**
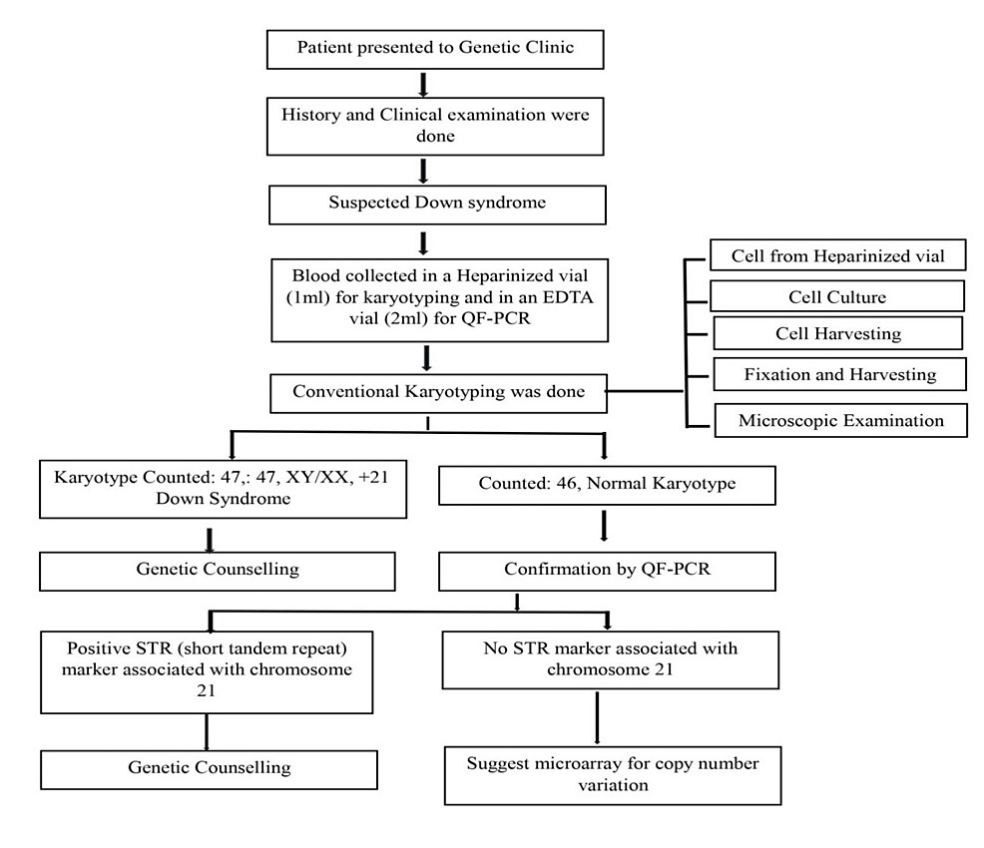
Flow chart depicting the methodology of the two diagnostic approaches

The A loading cocktail for capillary electrophoresis was produced by combining 2 µL of the size standard (560 SIZER ORANGE) with 100 µL of Hi-Di^TMb^ formamide. This was followed by filling a microwell plate or tubes (ABI310) with the required wells with 15 µL of the loading cocktail and placing it on the genetic analyzer [19]. After fragment length separation by capillary gel electrophoresis using the ABI GeneAmp® System 3500 set and Gene mapper Software, the results were graphed. Ratios were calculated using a spreadsheet. Figure 1 shows the flow chart illustrating the methodology of the two diagnostic approaches.

Statistical parameters

In our study, percentages were calculated to represent the proportion of participants exhibiting specific characteristics, providing insights into the distribution within the sample. Variables were summarized using mean ± standard deviation (SD) for the age, birth weight, and birth order, offering a clear depiction of central tendency and variability among the data. Differences between groups were not measured; therefore, p<0.05 or p<0.001 in the case of the null hypothesis was not considered.

Data Analysis

QF-PCR data were presented in graphical form. The ratios and results obtained were used to assess chromosome-specific STR markers [1].

Cost-effective approach

Karyotyping generally involves higher upfront costs due to the need for specialized equipment and skilled personnel. Reagents and consumables used in the process can also add to the overall cost. In QF-PCR, machines and reagents can be expensive as well, but the cost per test may be lower compared to karyotyping.

## Results

The sociodemographics of children with DS were assessed, and they had an average age of 25.95 ±23.55 months (Table 2); 55% (n=22) were female, and 45% (n=18) were male. Mothers' average age was 28 ±4.59 years, while that of fathers was 32.65 ±5.98 years. Of note, 50% (N=20) of the children were firstborns, 30% (n=12) were second born, and 20% (n=8) were third born. The children's average birth weight was 2.54 ±0.85 kg.

**Table 2:** Descriptive statistics of sociodemographic characteristics SD: standard deviation

Variables		Values (N=40)
Age of DS children, months, mean ±SD		25.95 ±23.55
Gender, n	Males	18
	Females	22
Mother's age, years, mean ±SD		28 ±4.59
Father's age, years, mean ±SD		32.65 ±5.98
Birth order, n	First	20
	Second	12
	Third	8
Birth weight, kg, mean ±SD		2.54 ±0.85

**Figure 2:**
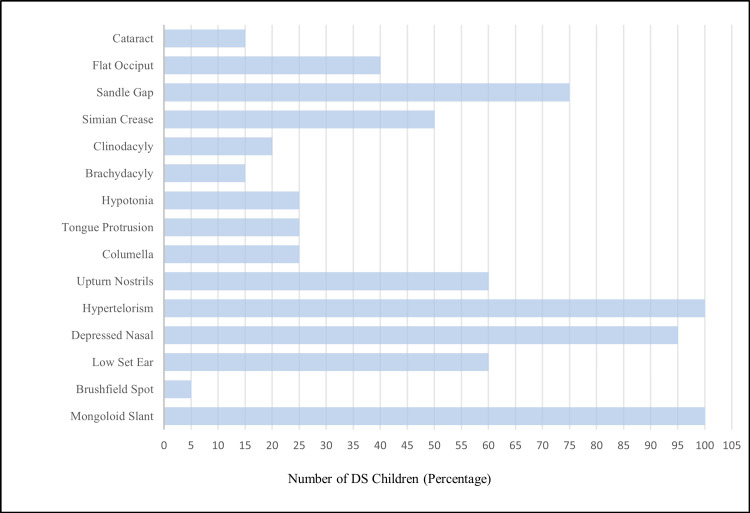
Clinical features of Down syndrome children

An analysis of the clinical features of DS children showed that children had mongoloid slant (n=40, 100%), brushfield spots (n=2, 5%), low-set ears (n=24, 60%), depressed nasal bridge (n=38, 95%), hypertelorism (n=40, 100%), upturn nostrils (n=24, 60%), columella (n=10, 25%), tongue protrusion (n=10, 25%), hypotonia (n=10, 25%), brachydacyly (n=6, 15%), clinodacyly (n=8, 20%), simian crease (n=20, 50%) sandle gap (n=30, 75%), flat occiput (n=16, 40%), cataract (n=6, 15%), wide anterior fontanelle (n=12, 30%), brachycephaly (n=24, 40%), and microcephaly (n=4, 10%) (Figure 2).

**Figure 3:**
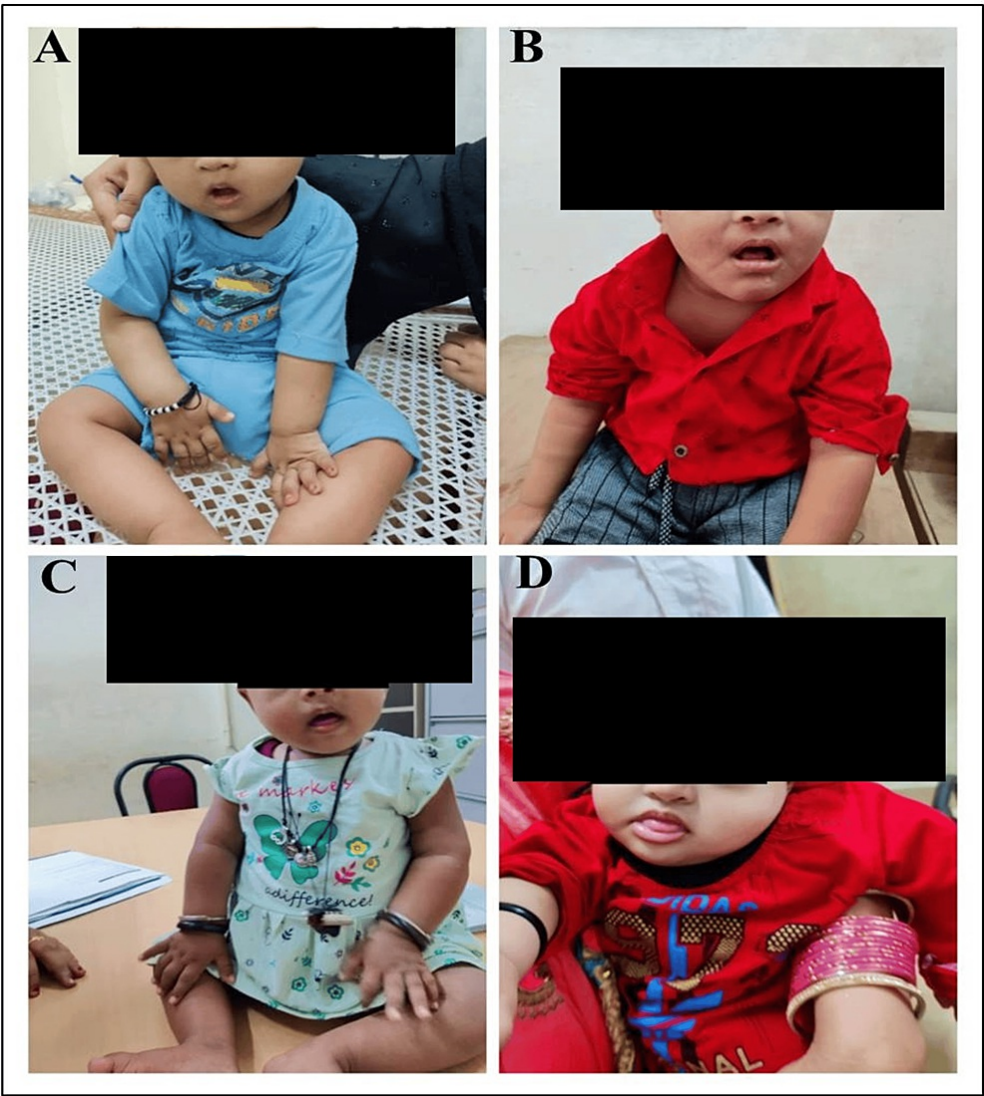
Facial features associated with Down syndrome A: flat facial profile; B: hypertelorism; C: nostril up turn; D: protruding tongue

Figure 3 illustrates certain facial features associated with DS. 

**Figure 4:**
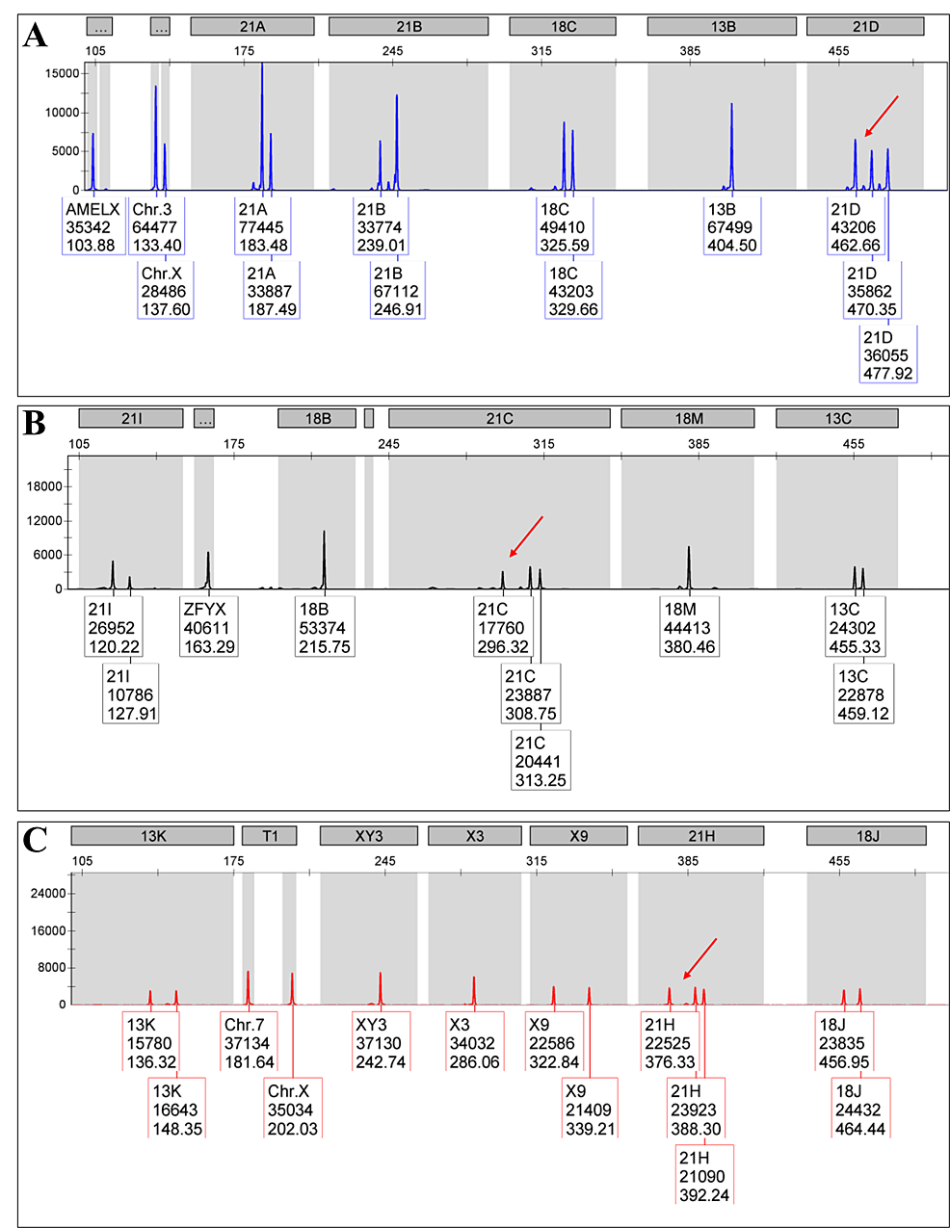
Illustrating a QF-PCR (electrophoretogram) report with three peaks, which indicate the detection of a 47, XX/XY trisomy 21 A: trisomy marker of 21D, characterized by a 1:1:1 ratio of STR D21S1444, located on 21q22.13 (red arrow); B: trisomy marker of 21C, characterized by a 1:1:1 ratio of STR D21S411, located on 21q22.3 (red arrow); C: trisomy marker of 21H, characterized by a 1:1:1 ratio of STR D21S1442, located on 21q21.3 (red arrow) QF-PCR: quantitative fluorescence polymerase chain reaction; STR: short tandem repeat

The karyotyping analysis revealed the presence of an additional chromosome 21 in both female and male children. Of note, 22 children with QF-PCR analysis identified specific STR markers associated with chromosome 21 (STR D21S1435, D21S11, D21S1411, and D21S1444). In another group, trisomy 21 with partial monosomy 18 was identified (two in number). In the former group, the karyotyping study showed an additional chromosome 21 but the QF-PCR analysis detected STR markers associated with both chromosome 21 (D21S1435, D21S11, D21S1411, and D21S1444) and chromosome 18 (D18S978, D18S535, GATA178F11). Mosaic trisomy 21 was observed in six children, which was evident in the karyotyping analysis (47, XX or XY, +21 cells, and 46, XX or XY). QF-PCR analysis did not detect any abnormality-specific STR markers. In the group with partial monosomy of chromosome 21 (three in number), the karyotyping analysis showed two different chromosomal configurations: one with the loss of chromosome 21 in 11 cells (20 observed) and the other with a deletion of chromosome 21 in seven cells (20 cells observed). QF-PCR analysis did not detect any abnormality-specific STR markers. The seven DS children exhibited no abnormality with conventional karyotyping and QF-PCR analyses. Figure 4 presents a QF-PCR (electrophoretogram) report. Table 3 details various abnormalities associated with the chromosomal aberrations after conventional karyotyping

**Table 3:** Different abnormalities associated with the chromosomal aberrations after conventional karyotyping QF-PCR: quantitative fluorescence polymerase chain reaction; STR: short tandem repeat

S. no.	Chromosome result	Karyotype	QF-PCR result of STR	Number of children
1	Trisomy 21	47, XX, +21 47, X.Y., +21	D21S1435, D21S11, D21S1411, D21S1444	22
2	Trisomy 21 with partial monosomy 18	47, XX or X.Y., +21	D21S1435, D21S11, D21S1411, D21S1444, D18S978, D18S535, GATA178F11	2
3	Mosaic trisomy 21	47, XX or X.Y., +21 with 46 normal	No detection of abnormality	6
4	Partial monosomy with 21	45, XX, - 21 [11] / 46, XX, del (21)	No detection of abnormality	3
5	No abnormality detected	46, XX or X.Y.	No detection of abnormality	7

As for the total time taken for the results of the karyotyping and QF-PCR, while conventional karyotype took 10-15 days to evaluate the final result, QF-PCR proved to be a rapid and more efficient method for diagnosing DS within three days. Table 4 illustrates the diagnostic approaches to the detection of clinically significant chromosome abnormalities.

**Table 4:** Diagnostic approaches to the detection of clinically significant chromosome abnormalities QF-PCR: quantitative fluorescence polymerase chain reaction

Observation	Description of karyotype	Description of QF-PCR
Technique	The traditional method for analyzing chromosomes by using microscopy	Molecular method based on PCR for detecting specific genetic markers
Detection scope	Able to identify a variety of chromosomal abnormalities, including significant structural alterations	Targeted approach, best suited for detecting specific genetic mutations or small-scale genetic variations
Sample type	Requires live cells obtained from sources like blood, amniotic fluid, or tissue samples	DNA extracted from various sources like amniotic fluid, blood, or other tissues
Diagnostic time	Typically takes several days to weeks to obtain results due to the time-consuming procedures involved	Results are ready in hours to days, which is quicker than karyotyping
Sensitivity	High sensitivity for detecting chromosomal abnormalities, especially large-scale changes	High sensitivity for detecting specific genetic markers targeted by PCR primers
Target abnormalities	Useful for identifying a variety of chromosomal diseases, including Turner syndrome, Down syndrome, and others	Appropriate for detecting certain genetic disorders like cystic fibrosis, thalassemia, and others
Interpretation complexity	Results require skilled personnel for sample preparation, analysis, and interpretation	Requires skilled personnel for primer design, PCR setup, and data interpretation

## Discussion

The present study compares the chromosomal abnormalities in DS using conventional karyotyping and molecular karyotyping (QF-PCR). The average age of the children included in the study was 25.95 ±23.55 months. Caregivers took more time to report, usually when the child was more than two years of age, as symptoms became more pronounced, e.g., developmental milestones not being achieved [20]. The mosaic DS indicates a diverse age distribution with varied clinical features, indicating the need for comprehensive care and support across different developmental stages [21]. The gender distribution among the children with DS was as follows: 45% male and 55% female. The higher incidence rate observed in females is in line with previous research [22]. The average age of the mothers in the study was 28 years, while that of fathers was 32.65 years. These findings indicate that DS can occur in children born to parents of various ages. However, it is worth noting that advanced maternal age is a known risk factor for DS.

Birth order analysis revealed that 50% of the children with DS were first-born, 30% were second-born, and 20% were third-born. These results suggest that the occurrence of DS is not influenced by birth order. However, larger studies comparing birth order patterns in children with DS and the general population could provide more insights into this relationship. The mean birth weight of the children with DS was 2.54 ±0.85 kg. While this falls within the normal range for birth weight, it is important to consider that DS can be associated with various physical and developmental challenges that may impact growth and weight gain in affected children [23]. Karyotyping analysis revealed the presence of an additional chromosome 21 in both XX and XY configurations, confirming the diagnosis of trisomy 21, the most common form of DS. This finding aligns with the well-established genetic basis of DS [24]. The QF-PCR findings show the relative quantity of each allele determined by the ratio of the peak heights or peak areas. An additional allele was detected as three peaks in a 1:1:1 ratio or as two peaks in a 2:1/1:2 ratio, indicating an additional chromosome (trisomy).

The study also revealed other chromosomal configurations associated with DS, such as trisomy 21 with partial monosomy 18, mosaic trisomy 21, and partial monosomy 21. While partial monosomy 18 was detected by QF-PCR, DS, however, could not be ascertained for mosaic 21 and partial monosomy 21. This highlights the complexity and heterogeneity of chromosomal abnormalities in DS, emphasizing the need for comprehensive genetic analyses to fully understand the spectrum of chromosomal variations associated with the disorder [25]. Hence, conventional karyotyping remains the gold standard for identifying DS in children. The QF-PCR method proved rapid and efficient compared to conventional karyotyping, which took a longer turnaround time. Applying QF-PCR in DS diagnosis can enhance the accuracy and timeliness of identifying chromosomal abnormalities, enabling earlier intervention and management strategies.

The focus on QF-PCR intended to emphasize its potential use in certain circumstances where quick and focused genetic information is required, for neonates with DS and mosaic with no clinical symptoms but with a high degree of suspicion [26]. Furthermore, the identification of chromosomal abnormalities through molecular karyotyping has important clinical implications. It allows for the detection of genetic variations beyond trisomy 21, enabling healthcare professionals to provide tailored interventions and support for individuals with DS and their families [27]. Thus QF-PCR is faster, less labor-intensive, and more cost-effective, making it a valuable tool in routine clinical practice [28]. It also has a higher resolution for detecting chromosomal abnormalities, allowing for a more accurate diagnosis [29]. Although Finnegan et al. found that the sensitivity of QF-PCR is low, in our study, the QF-PCR assay design was very accurate, and in all 28 patients, there were no instances of false-positive or false-negative results; the assay could detect chromosome 21 trisomy and even subtle clinical features of neonates but not with mosaic DS

The study's drawbacks include its limited sample size and narrow geographic scope, which may affect the generalization of its results to broader populations. Additionally, sociodemographic traits may vary among locations, which may have an impact on how broadly the results may be applied. This study focused on using QF-PCR to diagnose DS. However, future studies could explore other molecular techniques like FISH and CGH arrays to improve diagnostic capabilities and broaden the knowledge base about chromosomal abnormalities in DS.

This research article contributes to our understanding of the sociodemographic characteristics of children with DS and highlights the chromosomal abnormalities associated with the disorder. In comparison to conventional karyotyping, QF-PCR is a more cost-effective diagnostic method. It is a feasible choice in standard clinical practice because of its quicker turnaround time and lower labor intensity, which is important in healthcare settings with limited resources. However, the QF-PCR technique's function is limited to detecting specific genetic targets with known mutations and may not capture large-scale chromosome abnormalities or de novo changes [30].

## Conclusions

This study significantly contributes to the field of genetic testing for DS, offering healthcare professionals an efficient tool for rapid and reliable diagnosis, thereby benefiting individuals and families affected by this condition. The QF-PCR is a quicker and more reliable method, and, consequently, many samples may be diagnosed at once, and a rapid outcome is especially useful in lowering the time frame in the context of anxiety among parents surrounding reports. The results of molecular karyotyping (QF-PCR) and clinical features will hopefully aid in implementing need-based therapeutic interventions for DS children to improve their quality of life.
